# International Hahnemannian Association

**Published:** 1892-08

**Authors:** 


### INTERNATIONAL HAHNEMANNIAN ASSOCIATION MEETING OF 1892.

### The President's Address.

"The Mathewson," Narragansett Pier, R. I.
Tuesday, June 21st, 1892.

The thirteenth annual meeting of the International Hahnemannian Association was called to order by the President, James B. Bell, M. D., of Boston, Mass., at 11.30 A. M.

The first business was the reading of the address of the President, James B. Bell, M. D.

Dear Friends and Colleagues:

It gives me much pleasure to welcome you to this thirteenth annual meeting of our *most noble* Association.

I feel it to be no want of modesty thus to designate this society of men and women, whose high calling it is to heal the sick in the only pure and perfect method known to man, by unfailing devotion to the divine law of cure, and by a strict adherence to all the principles that grow out of that law.

Our patent of nobility, too, comes rather from our calling than from ourselves.

However unworthy we may feel ourselves to be, and however imperfect in our attainments, yet we are called, as an association, to be the official and international custodian of the great legacy left to our race by Hahnemann.

A sense, therefore, of responsibility to humanity should guide all our deliberations, discussions, and conclusions.

In casting about for some practical subject for the time-honored "President's Address," it occurred to me that, as there is in the commercial world an indispensable process, albeit a dusty, musty, and laborious one, called "taking account of stock," and which has to be done once a year or else all business would come to a chaos, so it might be well and profitable for us, after thirteen years of existence as a society, to follow the same process, and to take a candid and honest survey of our assets and liabilities as Hahnemannians, of the discouragements and encouragements for our cause, of the things that are for us, and the things that are against us, that we may know, as well as we can, exactly where we stand, and may be able, on the whole, to press more closely together, and to carry on the work more bravely and steadily than before.

The chief value of such meetings as these comes from the inspiration which they give, rather than from the actual amount of knowledge imparted or received.

Working, as many of us must, almost alone, and by ourselves, we need very much, at least once a year, to experience the fellowship of numbers. This is also the time and place, in the sunshine of our united enthusiasm, to face those things that sometimes almost appall us, when they come to us in the darkness and solitude of our work. Let us drag forth then all those things that ever seem the worst to us, and balance against them all that seems the best, and see if we shall not feel repaid for doing so.

We might find something easier and more poetic to do, but nothing, I think, more practical. The influence of this process, I trust, will be to increase our assets and diminish our liabilities.

As against us, we will notice—

1. *Our comparative fewness in numbers,* here and in all the world. Our Society numbers in active living members about 150, and it would be a generous estimate, I think, to double that number, as representing in the whole world all those who may be called true Hahnemannians or who are becoming such.

If we have patients going to other cities, especially in the West and South, how rarely can we recommend a physician to them, and if the patients are going to Europe or England, we know of but five or six men in the great cities to whom we can safely entrust them. We have also lost 23 by death, in thirteen years, which is a large number for us to lose.

The American Institute has about 1,300 members.

What have we to put down as an offset to these facts?

Well, first, I think, *quality*—as mentioned in my opening sentences.

As I look over the roll of members, I should not know where to search in any medical society for men and women of an equal average of ability and character.

If I may judge those whom I do not personally know by those whom I do know, I feel assured that the average of mental power, of intellectual honesty, and general professional acquirements; of those qualities fitted to deserve and gain the confidence of the better classes, in all communities, is much above that of all other medical societies.

I do not wish to seem invidious in this comparison, but I think we are entitled to this comfort in view of our small numbers.

I cannot properly cite the names of the living in proof of what I say, but glance over the names of our honored dead, those of whom we know most: Baer, Ballard, Bayard, Brown, the Ehrmans, Fellger, Foote, Gee, Gregg, Hawley, Keith, Kenyon, the Lippes, Pearson, Wells. Does not the memory of these men sustain my claim?

In numbers, too, our increase has been satisfactory. In 1880 we had 16 members. In 1883 we had 88, and all were living. In 1886 we had 117 living, and 5 had died. If we add, as we expect to do, about 15 members this year, our total of 165 will represent a gain of 1,000 per cent. in thirteen years. But as that is made up in the first years, of those who were already confirmed in the faith, it will be more fair to take the last five years, which will give us an average gain of 10 per cent. a year. This will double our number every ten years and fairly represent the direct influence of this Association, upon the homœopathic profession.

Our large death-rate, we trust, will now be reduced, as we add each year so many younger members.

Our third offset to the fewness of our numbers is the extent of our influence as an Association. Influence is, of course, a hard thing to measure, but I feel it safe to assert that if this Association had not been formed thirteen years ago, there would not have been one-half as many Hahnemannians as there are in this country to-day, nor would there be one journal of that type, one hospital, one dispensary, or one local *Organon* Society, or one post-graduate school. The parent society gives strength and courage to all these.

I have not had time to look over the recent Transactions of the American Institute, but I am informed by those faithful brethren who are good enough to attend the meetings of both societies that the papers and discussions have greatly improved in homœopathic tone, since this Association began to loom up as a power in the land. We know how much encouragement it is to the younger men and women who are coming on in the right path to find such a foster-mother ready to shelter and develop them.

2. But we must turn again to the other side of the ledger, and we must observe *the entrenched position of those who are opposed to us in almost everything but the name.* They have all the colleges and hospitals except an occasional teacher or staff attendant in some of them. They have all the journals but two. The graduates of those schools are rapidly increasing their numbers, and, as I much fear from what I can learn, deteriorating in Homœopathy, openly espousing and using the most antipathic and allopathic methods. The total number of so-called homœopathic physicians practicing in this manner in this country cannot be less than five or six thousand. They number many able, and I have no doubt, honest men of great influence in their respective communities.

If our patients or families find themselves in their localities, they will be strongly urged, if ill, to call the popular physician, and will probably do so, and get a dose of Morphine, Quinine, or Antipyrine before they get away. These men also have the control in most of the State and local societies, as well as in the Institute, and are thus extending their influence by their reports and discussions.

Per contra to the entrenched position of the "liberal" wing of our school, we have much to encourage us. Whether we will it or not, Homœopathy in the public estimation, in its legal and social status, stands as a unit; and the number of the physicians, the extent and work of the hospitals, schools, and dispensaries redound to the credit of all, and save us all from being pariahs, outcasts, and martyrs as were our predecessors of a generation or two ago.

Those representatives of our views who are attached to these institutions are doing good work so far as I know, and entirely unhindered by those with whom they are associated. I am sure that Dr. Wesselhœft and I have no fault to find with our colleagues in the Massachusetts Homœopathic Hospital, and we have often been pleasantly surprised to find the internes, who had been appointed solely on their merits, were also good Hahnemannians, and this without any effort or even knowledge on our part.

Three such, since graduated, are present at this meeting, or will be here.

I also expect that before we close this session, we shall have reports from several institutions already in existence, or about being organized, in addition to the Post-Graduate School of Philadelphia, and the Rochester Hargous Hahnemann Hospital. In many journals there are scattered articles and reports, which are sound and instructive in pure homœopathic principles.

We have now every reason to believe that if we are faithful and united we shall see these tangible and visible proofs of vitality and growth in the shape of new institutions and public services as the sure result.

3. We must turn again to the other side, and find our next count to be *the danger of disruption* even in our small body, and this danger never greater than now.

There have always been diverging elements and interests in this Association, personal, professional, temperamental, and perhaps geographical, and there is danger now that the centrifugal force may become greater than the centripetal.

The moon falls, as all falling bodies should, according to law, sixteen feet every second toward the earth, but it also travels just far enough in its orbit every second to find the earth curved just sixteen feet farther away.

If this balance of forces should be lost, something disastrous would happen.

Two bodies of equal weight, and moving with equal force, meeting at right angles, move off *together*, in a new direction. This resultant motion is *progress*. But if those bodies meet in opposing directions, the result is heat and destruction.

What do we find, now, upon the credit side of the ledger?

The spirit of union is stronger than the spirit of dissension. The spirit of brotherly confidence, respect, and even affection is stronger than that of doubt, discord, and censoriousness.

This has proved to be so for thirteen years, in spite of all tendencies to the contrary, and I believe it will be so still, only we must see to it that it is so.

The very cause of possible disruption is, to some degree, a favorable one. We cannot occupy the advanced position which we do, unless we are persons of strong individuality, deep convictions, courage, and strong wills.

We are not men and women of policy, but of principle. We are not lukewarm, but in hot and eager earnest in following our convictions. As these differ, the result will be a strong, centrifugal force.

But the balancing force is absolute unity in purpose and in fundamentals, and we must see that in all our discussions and decisions this balance is not lost.

Thus each of us, revolving in his own orbit, and together around the central sun of pure Hahnemannian principles, will form a little solar system of our own, with room for even a comet or two, with or without tails, and this will make an ideal I. H. A., both for the present and the future.

The resultant motion of heavy bodies, moving rapidly, and meeting thus at right angles will be progress and life, instead of injury and death.

I should be glad to utter a warning upon this point, the effects of which might last for many years—long after many of us shall have the star before our names upon the roll of members.

I am the more confident that this will be so, because I can conceive of no selfish or personal advantage to any of us in having it otherwise.

We all know the weakness of poor humanity upon the side of self, but we have not come into this Association for gain, whether of honor or of filthy lucre, or for the sake of having our own way. I have not heard that the Secretary or Treasurer has gotten rich out of it, or that any President, Chairman, or Censor has added in any way to his professional laurels by serving in the post to which we have called him, or that any of us have been gainers privately and professionally by our membership.

It may be different an hundred years hence, but I trust that the same simplicity and singleness of aim which brought us together will govern us to the end.

4. We come now to things which we have to meet in single combat. Let us group them together :.

*a.* The popular impression that the old school has been much improved by the new, so that it does not make much difference after all what kind of a doctor is employed if he is only one who "does not give much medicine."

*b.* The popular impression that anything is homœopathic which bears the name, whether it is a doctor or a nostrum from a "homœopathic" pharmacy.

*c.* Popular indifference to the danger of the prevalent abuse of fashionable drugs, Quinine, Antipyrine, Bromo-caffeine, etc., and the difficulty of convincing people of this danger.

*d.* The popular love of dosing in general with nostrums, tonics, bitters, purgatives, plasters, and salves.

*e.* The difficulty in even semi-homœopathic families in keeping patients from taking the great variety of sure-cure mineral waters, and applying "Pond's Extract" to every accessible part of the human body.

*f.* The difficulty of impressing people with the true pathology, especially in chronic diseases, and thereby leading them to give sufficient time for their cure.

*g.* The popular impression, increased by the multiplication of specialties, of the necessity of local treatment for all sorts of affections.

*h.* The occasional failure of our best efforts and most faithful labors, followed perhaps by the success, or apparent success of some allopathic brother in the same case.

These are enough for our present purpose.

Are there any encouragements to apply the only remedy to these difficulties, viz.—*a campaign of education?*

Can we overcome ignorance, prejudice, false conceptions, bad habits of thought and action, love of immediate ease and comfort, and the mighty weight of authority against us?

I think we can all see evidences of improvement along these lines.

In those families, and with those patients who have been long with us, we hear no more of those things. It is mostly with the new patients that we have these difficulties, and even with them it is often very interesting to notice how they gradually get rid of all these ideas and practices which trouble us.

I think we have every encouragement to persevere in giving the light of truth and wisdom upon all these points, but I want to pause right here to inquire if we are really doing it as we should, and how it ought to be done.

I spoke of the matter in my letter of acceptance and will now say further that I think we are very deficient in proper literature, simple, plain, brief-printed tracts or leaflets, fully explaining our philosophy, methods, purposes, and opposing the errors and practices of which I have spoken.

The year which marks the appearance of such a set of tracts or a variety of them will be a red-letter one in our calendar. Some of our most brilliant men or women must do the work, and let many of them try, and the fittest productions survive.

Let some of them deal with principles, some with abuses and errors, some with objections and difficulties.

Political parties and religious societies know well the value and importance of good expository literature, and make free and constant use of it. I am fully persuaded that the want of it is one of the greatest hindrances to our rapid and solid growth to-day. Personal interviews accomplish much, but quiet home reading will do a great deal more, impressing as it does the eye as well as the ear, and more lastingly. We need fear no rivals in this field for a long time. We could use thousands of such tracts every year. I recommend that a committee be appointed to consider the whole matter, to invite and solicit members to take up the work, to receive and pass upon such tracts as may be presented, to accept such as meet the requirements, and to arrange for their printing in a neat and attractive style by the Association for its benefit, and having its imprint and approval.

5. There is another subject which is difficult to classify because it seems to be both for and against us, as we look at it from different sides, but on the whole I place it upon the credit side of the ledger.

I refer to the subject of recruits, whence and how they come.

There is a law in the making of pure homœopathists which the evolutionist would call the survival of the fittest, and the Calvinist would call the election of grace.

We all recognize this law, I think, but it has not received sufficient attention.

It is important that we bear it in mind in order that our efforts may be wisely directed toward increasing our number. The fact seems to be that not every man or woman under the most favoring circumstances can comprehend, accept, or follow the teachings of Hahnemann in their purity and completeness.

It is useless to waste time with such. They may have great ability and gifts in other dirctions, but they can no more become followers of Hahneman than many of us, perhaps, could become disciples of Mozart or Beethoven.

They have no ear for that kind of music. Do we not call to mind prominent men of decided talent, great industry, and devotion to their work, and who began their career under the best instruction, even under Hering, Dunham, or the elder Wesselhœft, and who ran well for a time, as we know by records of their former work, but who have now gone entirely over to the other side, and have put forth teachings which, logically carried out, would wholly destroy Homœopathy as it seems to us.

I am not going to attack them for this. The trouble is in their make-up, and I only cite them as illustrations.

On the other hand, how many of us have come up out of the darkness of Egypt and across the waters of the Red Sea; have rowed up-stream and against the tide; have climbed from the shaded valley to the sunlit mountains, *always toward the light.*

Take our Sherbino or Cohen in the far Southwest, our Chapman or Martin on the Pacific, our Balch of the Northwest, or Holmes of Omaha. These men and many others, although cut off from the personal teaching and example of the fathers and brethren, yet how rapidly they have grown into the stature of full men in the faith.

We and they take no credit for this. We are made up that way. I doubt if any of us really could have been really mongrel homœopaths or allopaths any more than we could have been tight-rope performers or prize-fighters.

We should sooner have perished with disgust and disappointment if condemned to those delusive methods of healing the sick.

The law, then, seems to be that certain ones here and there will struggle toward the light as soon as their longing eyes perceive it. Our simple duty, then, is *to turn on the light*, to keep it bright, and to lead them gently to it.

That I conceive to be one of the chief functions of this Association.

6. I give you now one asset which covers all liabilities and leaves us a very large surplus. *We are in the right and we know it.* This may sound very bigoted, very narrow, very illiberal, and very opinionated to some, especially to our friends on the other side.

There are so many conflicting opinions upon politics, religion, and medicine that some would say that we can never know when we are right; that we must always be in a more or less hazy and uncertain condition upon all these subjects, just because other people are just as sure they are right as we are.

Now this view of things assumes that there is no such thing as certainty in anything, either because there is no abstract or concrete truth, or else that we poor human beings can never attain to it.

If this were true, our courts of justice, both civil and criminal, might as well go out of existence at once, just because both sides appearing there for trial are equally sure of the justice and righteousness of their cause. But for all this the judges and juries hear the cases, weigh the evidence, and listen to the arguments, and on the whole reach wise conclusions and render substantial, justice.

With minds properly poised, in the judicial attitude, and with like processes we may do the same.

Somebody must be in the right, and why not we?

Homœopathy has now been on trial about eighty-two years, presenting now a great mass of evidence in the form of arguments, reflection, and experience, and our verdict is unanimous and cannot be set aside.

I do not believe you can find anywhere a hundred and fifty men and women who are more solidly convinced of the certainty of their position than we are. No failures can daunt us. No imperfections of knowledge or means can discourage us. No taunts or sarcasms can affright us.

Turn by contrast to the other side, whether in ours or the other school. A wide reading of the journals and best textbooks will show you that our friends have some certainty in pathology and diagnosis, but when it comes to therapeutics they are practically agnostics. They *don't know* whether they are really doing any good or not, and the size of the "don't know" increases with their experience.

Sometimes, indeed, an occasional cure, with a similar remedy accidentally selected, will set them off all in a flutter to try and do it again in any case of like name, but with new and painfully disappointing results.

I am so thankful that I not only know that we are right, but I know it more surely every day, as the evidence accumulates from each day's work.

I do not forget that we sometimes have to say "we know," even almost through our tears, when after faithful work we see death at last the victor. But even here we can say this, as no others can, for the memory of many sure and swift successes of hard-fought battles won enable us to do this.

We have most of us shown the depth of our convictions, by trusting those dearest to us to the purest Homœopathy, in the most dangerous conditions, and I have no doubt that many of you, as well as I, have had your reward for this faith—in their speedy and perfect cure.

These convictions will doubtless be deepened by what we shall hear at this meeting.

We know we are right because we are founded upon the immutable laws of nature.

Here is a paradox: We *conquer* nature in proportion as we *obey* her laws.

All progress in the sciences is but a record of greater *obedience* to laws which were either unknown or but partially understood before.

All the recent progress in electrical science and art illustrates this, and there are yet whispers of yet much greater things in this department, just at hand, all to be yielded to better knowledge and better obedience.

Now we want to be just as broad and just as narrow as nature and nature's laws, and I can conceive of no other rational place for a rational man to occupy.

Bigotry, exclusiveness, or narrowness do not reside in the intellect—not in the strength or firmness of intellectual convictions, but in the *spirit toward others* with which convictions are held.

It will be a great gain to scientific progress in many directions when this fact is more fully apprehended.

